# Inhibitory Effects of Betulinic Acid on LPS-Induced Neuroinflammation Involve M2 Microglial Polarization via CaMKKβ-Dependent AMPK Activation

**DOI:** 10.3389/fnmol.2018.00098

**Published:** 2018-04-03

**Authors:** Chuwen Li, Chao Zhang, Hefeng Zhou, Yu Feng, Fan Tang, Maggie P. M. Hoi, Chengwei He, Dan Ma, Chao Zhao, Simon M. Y. Lee

**Affiliations:** ^1^State Key Laboratory of Quality Research in Chinese Medicine, Institute of Chinese Medical Sciences, University of Macau, Macau, China; ^2^School of Life Sciences, Beijing University of Chinese Medicine, Beijing, China; ^3^Department of Clinical Neurosciences, Wellcome Trust-MRC Cambridge Stem Cell Institute, University of Cambridge, Cambridge, United Kingdom

**Keywords:** AMP-activated protein kinase, betulinic acid, calmodulin-dependent protein kinase β, microglia polarization, neuroinflammation

## Abstract

In response to the microenvironment, microglia may polarize into either an M1 pro-inflammatory phenotype, exacerbating neurotoxicity, or an M2 anti-inflammatory phenotype, conferring neuroprotection. Betulinic acid (BA) is a naturally pentacyclic triterpenoid with considerable anti-inflammatory properties. Here, we aim to investigate the potential effects of BA on microglial phenotype polarization and to reveal the underlying mechanisms of action. First, we confirmed that BA promoted M2 polarization and inhibited M1 polarization in lipopolysaccharide (LPS)-stimulated BV-2 microglial cells. Then, we demonstrated that the effect of BA on microglial polarization was dependent on AMP-activated protein kinase (AMPK) activation, as evidenced by the fact that both AMPK inhibitor compound C and AMPK siRNA abolished the M2 polarization promoted by BA. Moreover, we found that calmodulin-dependent protein kinase kinase β (CaMKKβ), but not liver kinase B1, was the upstream kinase required for BA-mediated AMPK activation and microglial M2 polarization, via the use of both the CaMKKβ inhibitor STO-609 and CaMKKβ siRNA. Finally, BA enhanced AMPK phosphorylation and promoted M2 microglial polarization in the cerebral cortex of LPS-injected mice brains, which was attenuated by pre-administration of the AMPK inhibitor. This study demonstrated that BA promoted M2 polarization of microglia, thus conferring anti-neuroinflammatory effects via CaMKKβ-dependent AMPK activation.

## Introduction

Neuroinflammation is an inevitable and important pathological process involved in all types of damages to, and disorders of, the CNS (Ransohoff and Perry, 2009; Gemma, 2010; Dhama et al., 2015). Accumulating evidence has established activated microglia as major cellular elements of neuroinflammatory responses, executing specific immune functions to maintain physiological homoeostasis (Ransohoff and Perry, 2009; Gemma, 2010; Dhama et al., 2015). In response to various microenvironmental disturbances, microglia can be phenotypically polarized into a classical (pro-inflammatory; M1) or an alternative (anti-inflammatory; M2) phenotype (David and Kroner, 2011; Saijo and Glass, 2011; Joseph and Venero, 2013; Mantovani et al., 2013). Generally, activated M1 state microglia are characterized by increased levels of pro-inflammatory cytokines, including TNF-α, IL-1β, and IL-6, and the upregulation of iNOS, CD16, and CD68, etc., whereas M2 phenotype microglia have been shown to upregulate M2 phenotype markers, like Arg-1, CD206, Ym-1/2, and TGFβ, etc. (David and Kroner, 2011; Saijo and Glass, 2011; Joseph and Venero, 2013; Mantovani et al., 2013). Functionally, the M1 phenotype exacerbates neuronal injury and impedes cellular repair after CNS trauma and disorders. In contrast, the M2 microglia confers neuroprotection and promotes recovery and remodeling (David and Kroner, 2011; Saijo and Glass, 2011; Mantovani et al., 2013). Therefore, the combination of inhibiting the M1 phenotype and promoting the M2 stage is a potentially more viable strategy than mere inhibition of the M1 activation for treatment of neuroinflammatory disorders (Hu et al., 2015). However, there are few compounds reported to regulate microglia polarization toward the M2 phenotype (Lu et al., 2010; Zhou et al., 2014).

Current evidence indicates that cellular energy metabolism not only participates in inflammatory responses but also regulates the conversion of functional phenotypes of microglia (O’Neill and Hardie, 2013). The AMPK is an evolutionarily conserved intracellular energy metabolism sensor that has a central role in maintaining energy homeostasis (Amato and Man, 2011; O’Neill and Hardie, 2013). The crystal structure of AMPK has a catalytic α subunit, as well as two regulatory (β and γ) subunits. It is well known that AMPK activation is dependent on the phosphorylation of α-subunit (Thr172 residue), which is mainly regulated by LKB1 or calcium/CaMKKβ in response to energy deprivation and abnormal levels of intracellular Ca^2+^, respectively (Amato and Man, 2011; O’Neill and Hardie, 2013). In fact, there are also reports indicating that AMPK activators could significantly inhibit M1 phenotypical inflammatory responses, thus protecting against cellular and tissue damage (Sag et al., 2008; Bai et al., 2010). Moreover, the activation of AMPK signal pathways has been shown to promote macrophage/microglia polarization toward the M2 stage, thus conferring anti-inflammatory effects (Mounier et al., 2013; O’Neill and Hardie, 2013). On the other hand, it has been demonstrated that the anti-inflammatory properties of some widely known anti-inflammatory agents are associated with the activation of the AMPK signaling pathway (Lu et al., 2010; Lee and Kim, 2014; Mancuso et al., 2014; Zhou et al., 2014; Hussein et al., 2015; Liu et al., 2016; Velagapudi et al., 2017). Therefore, AMPK has been considered as an attractive target for the treatment of inflammatory disease (Amato and Man, 2011; O’Neill and Hardie, 2013).

Betulinic acid [3β-hydroxy-lup-20(29)-en-28-oic acid] is a naturally pentacyclic triterpenoid obtained from the outer bark of several tree species, mainly white-barked birch trees (de Melo et al., 2009). This compound has been reported to possess various kinds of biological properties, including anti-inflammatory, anticancer, antimalarial, and antiplatelet activities, etc. (Lee et al., 2011; Wan et al., 2013; Jingbo et al., 2015; Ju et al., 2015; Lingaraju et al., 2015; Kim et al., 2016; Laavola et al., 2016). The anti-inflammatory effect of BA is well recognized in several different models (Saaby and Nielsen, 2012; Wan et al., 2013; Ju et al., 2015; Lingaraju et al., 2015; Kim et al., 2016; Laavola et al., 2016). First, BA could inhibit LPS-stimulated release of inflammatory cytokines in RAW 264.7 macrophages and protect against animal death and tissue damage during LPS/polymicrobial-induced sepsis (Lingaraju et al., 2015; Kim et al., 2016). Also, BA was found to suppress inflammation via activation of PPAR-γ in human osteoarthritis chondrocytes (Jingbo et al., 2015). In addition, BA was reported to decrease LPS-induced matrix metalloproteinase-9 expression via blocking the nuclear translocation of nuclear factor-κB in microglial cells (Lee et al., 2011; Wan et al., 2013). Recently, several reports also demonstrated that BA could activate AMPK pathways *in vitro* and *in vivo* (Jin et al., 2016; Silva et al., 2016; Xie et al., 2017). It is worth noting that BA-induced CaMKKβ-dependent AMPK activation was reported to confer protective actions in endothelial cells and in a non-alcoholic fatty liver disease mouse model (Jin et al., 2016). However, whether BA could promote microglial polarization to M2 phenotype and whether AMPK activation is involved in the anti-neuroinflammatory effects of BA have not been investigated.

In the current study, we aimed to test the potential effects of BA in microglia M2 polarization and to reveal the underlying mechanisms of action. We found that BA induced AMPK activation and promoted microglia polarization toward the M2 phenotype, thus inhibiting neuroinflammation. Furthermore, we demonstrated that CaMKKβ-dependent AMPK activation is required for BA-induced microglia M2 polarization.

## Materials and Methods

### Chemicals

The BA (purity > 98%) and LPS (*Escherichia coli* serotype 055: B5) were supplied by J&K Scientific (Beijing, China) and Sigma-Aldrich (St. Louis, MO, United States), respectively. The compounds C, metformin, STO-609, and A769662 were obtained from Selleck Chemicals (Shanghai, China). RPMI 1640, DMEM, OPTI medium, FBS, penicillin, streptomycin, RNA iMAX Kit, DAPI solution, and BCA kit were purchased from Thermo Fisher Scientific (Carlsbad, CA, United States). NO Colorimetric Assay Kit was purchased from BioVision (Milpitas, CA, United States). The ELISA Ready-SET-Go Kits for TNF-α and IL-10 were purchased from eBiosciences (San Diego, CA, United States). High Pure RNA Isolation Kit, Transcriptor First Strand cDNA Synthesis Kit, and FastStart Universal SYBR Green Master Reagents were purchased from Roche Applied Science (Mannheim, Germany). AMPKα, p-AMPK (Thr172), LKB1, p-LKB1 (Ser428), p-CaMKKβ (Ser511), ACC, p-ACC (Ser79), β-actin, GAPDH, and HRP-linked secondary antibody were purchased from Cell Signaling Technology (Beverly, MA, United States); iNOS, CaMKKβ, and Arg-1 were purchased from Invitrogen (Carlsbad, CA, United States); CD68, CD206, and YM1/2 were purchased from Abcam (Cambridge, MA, United States). The donkey anti-goat, Alex Fluor 488, and donkey anti-rabbit, Alex Fluor 647, secondary antibodies were obtained from Jackson ImmunoResearch Laboratories (West Grove, PA, United States). ECLplus Western Blotting Detection Reagents Kit was purchased from GE Healthcare (NJ, United States). All other chemicals and solvents were of molecular biology grade.

### Cell Culture

BV-2 cells, a widely used immortalized murine microglial cell line, were obtained from Kunming Cell Bank of Type Culture Collection, Kunming Institute of Zoology (Saleppico et al., 1996; Wu et al., 2015). HeLa cells were purchased from the American Type Culture Collection (Rockville, MD, United States). BV-2 cells and HeLa cells were cultured in RPMI 1640 and DMEM, respectively. The medium was supplemented with 10% FBS, 100 U/mL penicillin, as well as 100 μg/mL streptomycin, in an atmosphere of 95% air and 5% CO_2_ at 37°C. Cells were then passaged three times per week and used when the confluence reached about 80%.

### Cell Viability Assay and Morphological Analysis

Cell viability was assessed by the MTT assay. Briefly, cells were seeded in 24- or 96-well culture plates and received with indicated treatments. After that, cells were incubated with MTT and finally the absorbance at 570 nm was measured using a Flexstation 3 Microplate Reader (Molecular Devices, CA, United States). For morphological analysis, cells were washed with pre-warmed medium without phenol red, then observed, and imaged using the Olympus IX73 microscope system (Olympus, Tokyo, Japan).

### Experimental Animals and Protocols

Male C57BL/6 mice (25–30 g, about 8 weeks old) were obtained from The Chinese University of Hong Kong. Animals were maintained specific pathogen-free conditions with a 12 h light/12 h dark cycle, under standard temperature and humidity, and provided with regular daily diets and clean water *ad libitum*. All animal experiments were performed according to the National Institutes of Health Guide for the Care and Use of Laboratory Animals with prior approval from the Institutional Animal Ethical Committee (UMARE-007-2016 and UMARE-AMEND-042).

The animals were randomly divided into four groups (*n* = 9): vehicle group, LPS group, LPS+BA (30 mg/kg) group, and BA (30 mg/kg) group. BA was initially dissolved in 5% (v/v) Tween 80 and then further suspended in normal saline. BA (30 mg/kg, i.p.) was given once daily between 10:00 and 11:00 for 3 days. The administration route and dose of BA were set based on previous studies (Lingaraju et al., 2015). Treatment alone with BA did not reduce food intake or any apparent toxicity in animals. On day 4, the animals were injected intraperitoneally with LPS (1 mg/kg) 1 h after the last drug administration (Henry et al., 2008; Wu et al., 2015; Xu et al., 2015); 6 h after the LPS stimulation, the animals were anesthetized and immediately intracardially perfused with normal saline. Finally, the whole brain was collected, and the cerebral cortex was separated for additional experiments.

### NO Assay

Microglial production of NO was assessed by measuring the accumulated nitrite released into culture media. Briefly, BV-2 microglial cells were stimulated with LPS for 6 or 24 h. Then, culture media were collected and analyzed via a NO Colorimetric Assay Kit according to the manufacture’s protocol.

### Enzyme-Linked Immunosorbent Assay (ELISA)

The TNF-α and IL-10 release into conditioned media collected from BV-2 microglia cells were assessed by ELISA Ready-SET-Go Kits (eBiosciences, San Diego, CA, United States). The levels were quantified following the manufacturer’s protocols.

### Quantitative PCR (qPCR) Assay

Total RNA was extracted using the High Pure RNA Isolation Kit according to the manufacturer’s protocol. Isolated RNA was then reverse-transcribed into cDNA using the Transcriptor First Strand cDNA Synthesis Kit following the standard protocol. The qPCR assay was conducted using Fast Start Universal SYBR Green Master Reagents with the Applied Biosystems 7900 HT Fast Real-Time PCR System (Applied Biosystems, Inc., Foster City, CA, United States). The amplification parameters used here were 50°C for 2 min then 95°C for 10 min, followed by 40 cycles of 95°C for 15 s and 60°C for 30 s. Each sample was analyzed in triplicate, and the relative expression of mRNA was calculated after normalization to β-actin. All primer sequences used are listed in Supplementary Table 1.

### Small Interfering RNA (siRNA) Transfection

At 24 h after plating, BV-2 cells with a confluence of 70–80% were transfected with scrambled control siRNA, AMPKα siRNA, or CaMKKβ siRNA (GenePharma, Shanghai, China). In brief, RNA iMAX and siRNA were pre-mixed in OPTI medium according to manufacturer’s protocols and then added into cultured cells; 24 or 36 h after transfections, OPTI medium was replaced by standard DMEM medium with FBS for further experiments. All siRNA sense strands used are listed in Supplementary Table 2.

### Western Blot Analysis

BV-2 cells and mouse brain cortex samples were lysed in RIPA lysis buffer (Beyotime, Shanghai, China). Then, lysed sample homogenates were centrifuged at 13,500 × rpm at 4°C for 20 min. After centrifugation, the supernatants were collected and the protein concentration was assessed by the BCA kit. Aliquots of protein samples were resolved by SDS-PAGE (7.5–15%) and transferred to PVDF membranes. Membranes were blocked with 5% BSA, followed by incubation at 4°C overnight with diluted primary antibodies. Membranes were washed and incubated with HRP-anti-rabbit secondary antibody for 1 h at room temperature. Finally, protein bands were visualized using an ECLplus Western Blotting Detection Reagents kit. Membranes were analyzed using a Bio-Rad ChemiDoc XRS Imaging System with Quantity One Software (version 4.5.2) (Bio-Rad, Hercules, CA, United States).

### Immunohistochemistry

For BV-2 microglia staining, cells were washed, fixed, permeabilized, and blocked. After that, cells were incubated at 4°C overnight with the primary antibodies. The next day, cells were incubated secondary antibodies and DAPI. Finally, the samples were imaged using the In Cell Analyzer 2000 system (GE Healthcare).

For mouse brain staining, serial coronal cryosections (20 μm thick) of cortex of each brain were prepared according to the previous protocol. Briefly, after being fixed, washed, permeabilized, and blocked, sections were incubated with primary antibody. After that, sections were further incubated with secondary antibody and DAPI solution. Sections were imaged by a confocal microscope TCS SP5 (Lecia, Solms, Germany). Cell numbers were calculated by counting of per random microscopic field in a blinded fashion. Data are expressed as the percentage of CD16/32^+^ or CD206^+^ cells to the Iba-1^+^ cells.

### Phagocytosis Assay

Phagocytic property was evaluated based on the uptake of IgG FITC-conjugated latex beads (Phagocytosis Assay Kit; Cayman Chemical, Ann Arbor, MI, United States) as per the manufacturer’s protocol. Then, cells were observed and imaged using the In Cell Analyzer 2000 system. The fluorescence intensity of the cells was analyzed using Image J software and then normalized to the untreated cells (National Institutes of Health, United States).

### Graphing and Statistical Analysis

Statistical analyses were performed using GraphPad Prism software (version 6.0; GraphPad Software, Inc., San Diego, CA, United States), and data are represented as means ± standard error of the mean (SEM). Statistical analysis of differences between two groups was done using the independent-samples’ *t-*test and one-way or two-way ANOVA with Bonferroni’s correction applied was used for multiple group comparisons. Pearson’s correlation coefficient was used for correlation analyses. *P <* 0.05 was considered as statistically significant in all analyses.

## Results

### BA Prevented LPS-Induced M1 Microglial Activation and Promoted Microglial Polarization Toward the M2 Phenotype in BV-2 Microglial Cells

**FIGURE 1:**
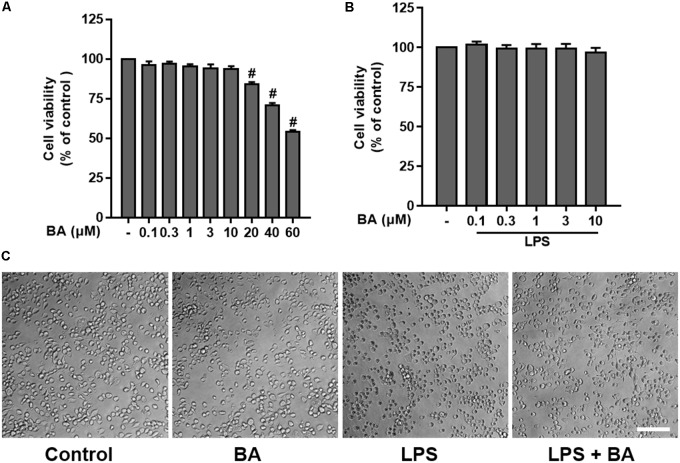
Effects of betulinic acid (BA) on the cell viability and morphological change of BV-2 microglial cells, with or without lipopolysaccharide (LPS) stimulation. BV-2 microglial cells were treated with BA with or without incubation of LPS (100 ng/mL) for 24 h. **(A)** Effects of BA alone on the cell viability. **(B)** Effects of BA on the cell viability with LPS stimulation. **(C)** Effects of BA on morphological change, with or without LPS stimulation. Scale bar, 100 μm. Data are presented as means ± SEM of three independent experiments in triplicate. Control group was untreated cells. ^#^*P <* 0.01, versus control group; ^∗^*P <* 0.05 and ^∗∗^*P <* 0.01, versus LPS-treated group.

In order to estimate the range of effective concentrations, we first measured the effects of BA, from 0.1 to 60 μM, on the cell viability of BV-2 cells using the MTT assay. The results showed that BA alone at concentrations lower than 10 μM did not cause any detectable cytotoxicity in either BV-2 cells, with or without LPS stimulation for up to 24 h (**Figure 1**, all *p* < 0.01 versus LPS-treated group). Therefore, concentrations of 0.3, 1, 3, and 10 μM were used in the following investigations.

In addition, the morphological changes of BV-2 following treatment with BA or LPS were observed. As shown in **Figure 1C**, the cells exhibited small soma with distal arborization, the typical ramified morphology of resting microglia in the control untreated group. BA (10 μM) alone treatment did not induce any morphological changes of BV-2 cells. Following treatment with LPS, the microglia became fewer and shorter branches with a greatly enlarged cell body, the characteristic shapes of activated microglia (Cheon et al., 2017). However, treatment with BA attenuated the LPS-induced morphological changes in BV-2 microglia.

**FIGURE 2:**
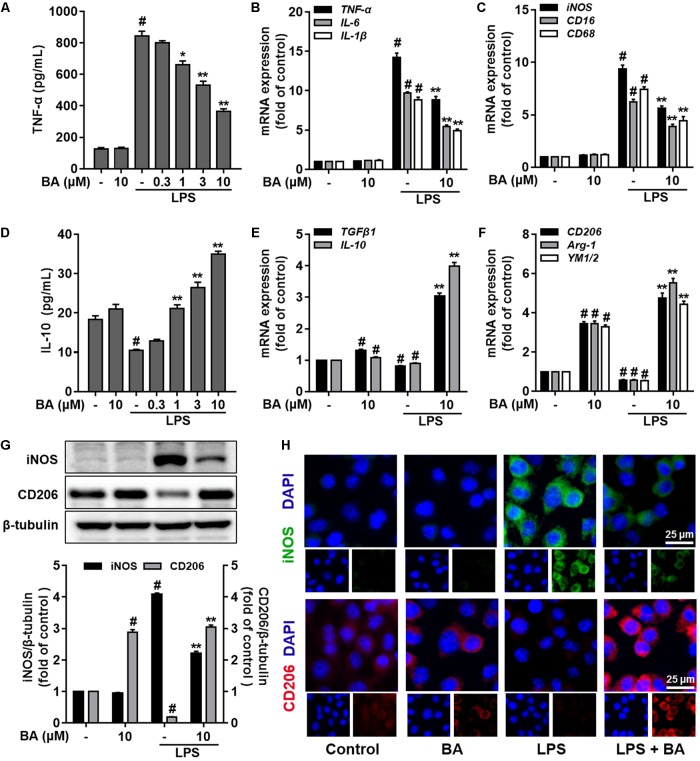
BA prevented LPS-induced M1 microglia activation and promoted microglia polarization to the M2 phenotype in BV-2 microglial cells. Cells were treated with BA for 1 h, followed by LPS stimulation for 6 or 24 h. The cytokine level was determined by ELISA. The mRNA expression level was determined by qPCR assay. **(A)** BA inhibited LPS-induced TNF-α release into the culture media of BV-2 cells. **(B,C)** BA inhibited LPS-induced mRNA expression TNF-α, IL-1β, IL-6, iNOS, CD16, and CD68. **(D)** BA enhanced IL-10 release into culture media. **(E,F)** BA prevented the LPS-induced downregulation of TGFβ1, IL-10, CD206, Arg-1, and YM1/2expression. **(G,H)** BA significantly decreased the iNOS expression, whereas increased the CD206 expression in BV-2 cells, evidenced by Western blot assay and immunofluorescence staining. Scale bar, 25 μm. Data are presented as means ± SEM of three independent experiments in triplicate. Control group was untreated cells. ^#^*P <* 0.01, versus control group; ^∗^*P <* 0.05 and ^∗∗^*P <* 0.01, versus LPS-treated group.

Then, we evaluated the role of BA in the LPS polarization of BV-2 microglial cells. First, as illustrated in **Figure 2**, LPS stimulation drastically induced M1 pro-inflammatory polarization, as evidenced by the induction of the M1 pro-inflammatory cytokine TNF-α (**Figure 2A**) and reduction of the M2 anti-inflammatory cytokine IL-10 (**Figure 2D**) in BV-2 microglial cells (all *p* < 0.01 versus the control group). BA conferred little effect on microglia polarization in the resting condition, while BA significantly suppressed LPS-induced release of TNF-α (**Figure 2A**) in a dose-dependent fashion (all *p* < 0.01 versus the LPS-treated group). Conversely, BA increased IL-10 release in LPS-stimulated BV-2 microglial cells in a dose-dependent manner (**Figure 2D**, all *p* < 0.001 versus the LPS-treated group). Furthermore, BA significantly reduced mRNA expression of pro-inflammatory cytokines, such as TNF-α, IL-6, and IL-1β (**Figure 2B**) and pro-inflammatory mediators, like iNOS, CD16, and CD68 (**Figure 2C**), in LPS-activated BV-2 microglial cells. Next, we found that the LPS-induced decreased mRNA expression levels of M2 marker genes, including IL-10, TGFβ1, CD206, Arg-1, and Ym1/2, were also significantly increased by BA (10 μM) in BV-2 microglia (**Figures 2E,F**, all *p* < 0.01 versus LPS-treated group). Moreover, LPS stimulation decreased the expression of CD206 at the protein level (**Figure 2G**), whereas BA incubation prevented this tendency and increased the level of CD206 to ∼15-fold that of the LPS-treated group in BV-2 (all *p* < 0.01). In addition, BA decreased the protein expression of iNOS to ∼0.5-fold that of the LPS-treated group in BV-2 (**Figure 2G**, *p* < 0.01). To conclude, BA prevented LPS-induced M1 microglial polarization and promoted the M2 phenotype.

### BA Enhanced AMPK Phosphorylation in BV-2 Microglial Cells

**FIGURE 3:**
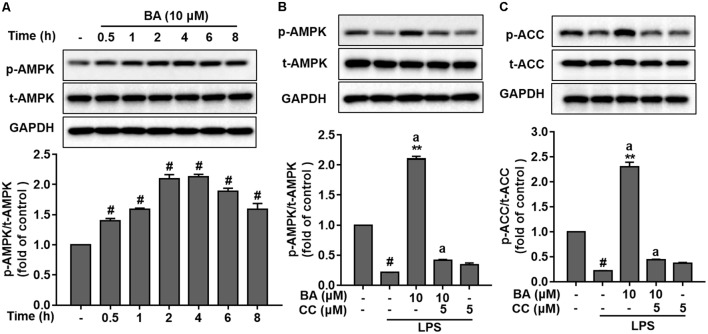
BA enhanced AMPK activation in BV-2 microglial cells. Cells were pretreated with or without AMPK inhibitor compound C (CC) for 0.5 h and then treated with BA alone or BA and LPS. **(A)** BA increased AMPK phosphorylation in BV-2 cells without LPS stimulation. **(B,C)** The AMPK inhibitor CC inhibited BA-enhanced AMPK and ACC phosphorylation. Data are presented as means ± SEM of three independent experiments in triplicate. Control group was untreated cells. ^#^*P <* 0.01, versus control group; ^∗^*P <* 0.05 and ^∗∗^*P <* 0.01, versus LPS-treated group. Two columns sharing the same letter are significantly different (*P <* 0.05).

It is well known that AMPK activation is dependent on the phosphorylation of Thr172 in the protein loop (Amato and Man, 2011; O’Neill and Hardie, 2013). Of note, in LPS stimulation-free BV-2 cells, BA (10 μM) significantly elevated AMPK phosphorylation; the elevation was found at 0.5 h, peaked at about 2 h, and lasted for more than 8 h, after BA treatment (**Figure 3A**, *p* < 0.01 versus control group). Previous reports indicated that LPS-stimulated macrophages displayed a reduced AMPK activation (Sag et al., 2008). Consistently, we found that LPS markedly decreased the phosphorylation of AMPK in microglia after LPS stimulation (**Figure 3B**, *p* < 0.01 versus control group). However, BA treatment reversed the LPS-inhibited AMPK phosphorylation (**Figure 3B**, all *p* < 0.01 versus control group). More relevantly, BA-enhanced AMPK and ACC phosphorylation was remarkably weakened by compound C, a well-established AMPK inhibitor, in BV-2 cells (**Figure 3B**). To further verify that BA enhanced AMPK activity, we tested the role of BA in the phosphorylation (Ser-79) of ACC, a well-established substrate of activated AMPK. BA significantly increased levels of phosphorylated ACC in BV-2 cells (**Figure 3C**), which clearly indicated enhancing effects of BA on AMPK activity. In conclusion, we found that BA could promote AMPK phosphorylation in microglial cells.

### AMPK Activation Was Required for BA-Mediated M2 Microglial Polarization in BV-2 Microglial Cells

**FIGURE 4:**
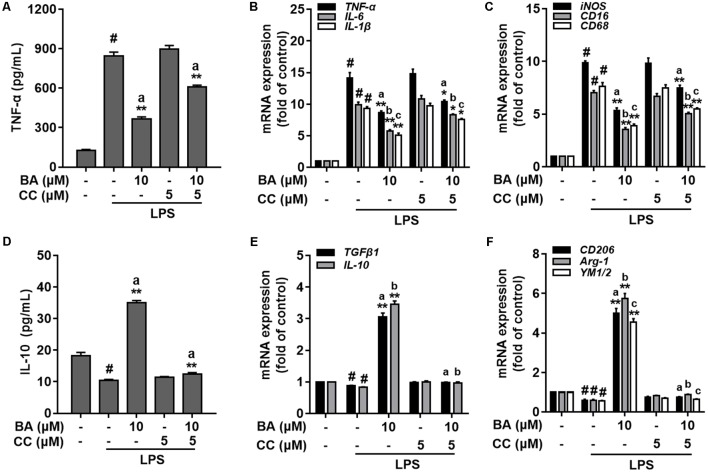
AMPK activation is involved in BA-mediated microglial M2 microglia polarization in BV-2 microglial cells. BV-2 cells were pretreated with AMPK inhibitor compound C (CC) for 0.5 h, and then with BA for another 1 h, followed by exposure to LPS stimulation for 6 or 24 h. **(A)** AMPK inhibitor attenuated BA inhibition of LPS-induced TNF-α production. **(B,C)** The AMPK inhibitor reversed BA inhibition of LPS-induced mRNA expression of TNF-α, IL-1β, IL-6, iNOS, CD16, and CD68. **(D)** The AMPK inhibitor abolished BA-enhanced IL-10 release into culture media. **(E,F)** The AMPK inhibitor abolished BA-enhanced mRNA expression of TGFβ1, IL-10, CD206, Arg-1, and YM1/2. Data are presented as means ± SEM of three independent experiments in triplicate. Control group was untreated cells. ^#^*P <* 0.01, versus control group; ^∗^*P <* 0.05 and ^∗∗^*P <* 0.01, versus LPS-treated group. Two columns sharing the same letter are significantly different (*P <* 0.05).

**FIGURE 5:**
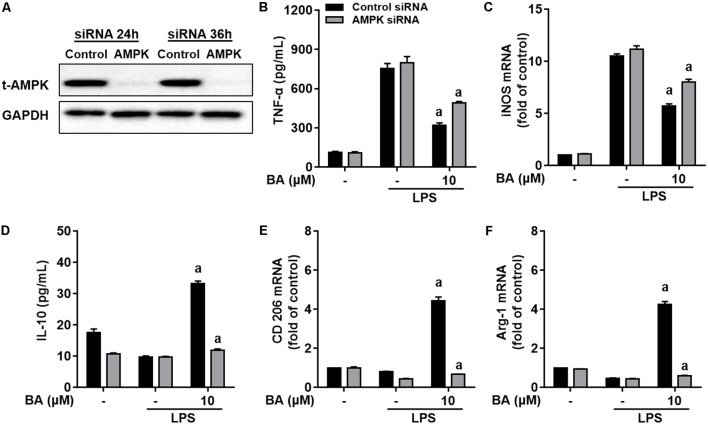
Knockdown of AMPK by siRNA attenuated BA-mediated microglia polarization in BV-2 microglial cells. Cells were first transfected with 40 nM AMPKα siRNA for 24 h, and then treated with BA for 1 h, followed by exposure to LPS stimulation for 6 or 24 h. **(A)** Transfection for 24 h was able to decrease the expression of AMPKα protein. **(B,C)** AMPKα knockdown decreased BA-mediated inhibition of TNF-α production, and mRNA expression of TNF-α and iNOS in LPS-stimulated BV-2 cells. **(D–F)** AMPKα knockdown attenuated BA-enhanced IL-10 release and mRNA expression of CD206 and Arg-1. Data are presented as means ± SEM of three independent experiments in triplicate. Control group was that treated with control siRNA but not BA or LPS. Two columns sharing the same letter are significantly different (*P <* 0.05).

To investigate the role of BA-induced AMPK activation in microglial polarization, we then tested whether AMPK inhibition by pharmacological inhibition or AMPK-specific siRNA could block the M2 microglial polarization caused by BA. First, BA-induced inhibition of TNF-α expression, and gene expression of TNF-α, IL-6, IL-1β, iNOS, CD16, and CD68 were in part attenuated by the AMPK inhibitor, compound C, in BV-2 cells (**Figures 4A–C**, all *p* < 0.01 versus the LPS-treated group). Moreover, compound C entirely abolished BA-mediated upregulation of IL-10, TGFβ1, CD206, Arg, and Ym1/2 in LPS-treated BV-2 (**Figures 4E,F**, all *p* < 0.01 versus the BA and LPS-treated group). In addition, compound C decreased IL-10 protein expression levels, and antagonized the enhanced expression by BA treatment in BV-2 cells (**Figure 4D**, *p* < 0.01 versus the BA and LPS-treated group). Then, the AMPK siRNA was used to knockout AMPK in BV-2 microglial cells; as expected, AMPK protein expression was significantly decreased at 24 and 36 h after transfection (**Figure 5A**). Therefore, 24 h after transfection was selected in this study. AMPK knockdown partially abolished BA-mediated inhibition of LPS-induced production of TNF-α, as well as iNOS mRNA level (**Figures 5B,C**, both *p* < 0.01 versus control siRNA group). Consistent with the results obtained using the AMPK inhibitor, AMPK knockdown by siRNA totally reversed BA-enhanced IL-10 release and expression of CD206 and Arg-1, indicating that the BA-induced M2 microglial polarization was significantly suppressed by AMPK gene silence in BV-2 microglial cells (**Figures 5D–F**, all *p* < 0.01 versus the control siRNA group). Collectively, we showed that BA-promoting effects on M2 phenotype polarization were due in part to BA-induced AMPK activation in microglial cells.

### CaMKKβ Was the Upstream Kinase Involved in BA-Induced AMPK Activation in BV-2 Microglial Cells

**FIGURE 6:**
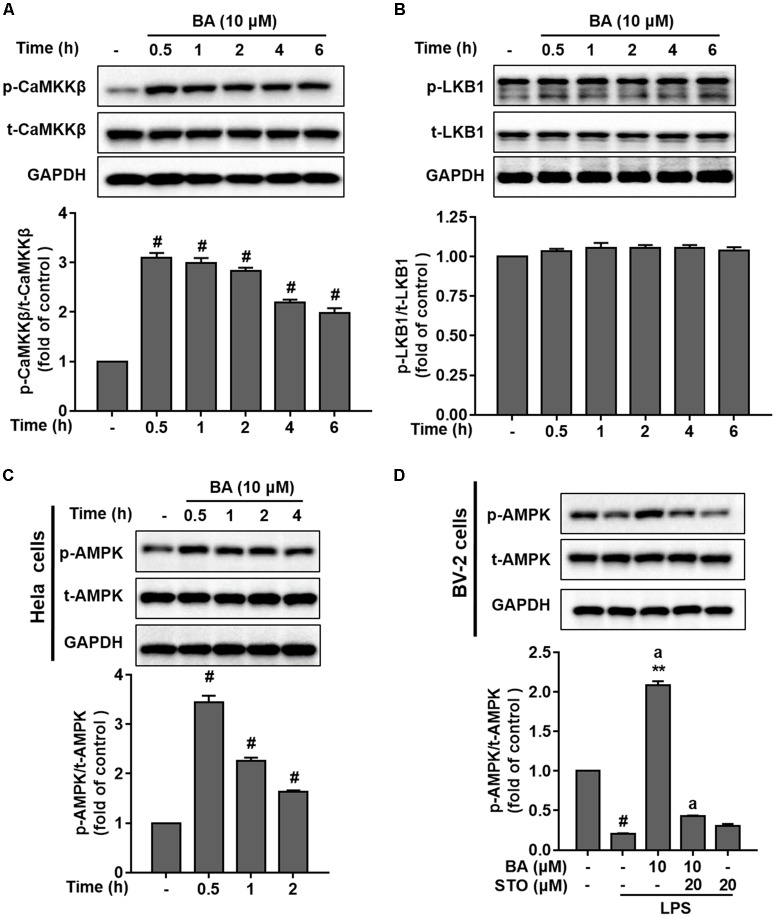
BA activated AMPK in a CaMKKβ-dependent manner in BV-2 microglia cells. Cells were pretreated with or without CaMKKβ inhibitor STO-609 (STO) for 0.5 h and then treated with BA alone or BA and LPS. **(A)** BA time-dependently increased CaMKKβ phosphorylation in BV-2 cells. **(B)** BA had no effect on LKB1 phosphorylation in BV-2 cells. **(C)** BA was able to increase the level of AMPK phosphorylation in HeLa cells. **(D)** STO attenuated BA-induced AMPK activation in BV-2 microglia. Data are presented as means ± SEM of three independent experiments in triplicate. Control group was untreated cells. ^#^*P <* 0.01, versus control group; ^∗^*P <* 0.05 and ^∗∗^*P <* 0.01, versus LPS-treated group. Two columns sharing the same letter are significantly different (*P <* 0.05).

AMP-activated protein kinase is mainly activated by two major upstream kinases, CaMKKβ and LKB1, in response to energy deprivation and abnormal levels of intracellular Ca^2+^, respectively (Amato and Man, 2011; O’Neill and Hardie, 2013). To identify which kinase was required for BA-induced AMPK activation, we first tested the effects of BA on the phosphorylation of CaMKKβ and LKB1. BA (10 μM) significantly activated CaMKKβ (**Figure 6A**), but not LKB1 (**Figure 6B**) in BV-2 microglia cells (all *p* < 0.01 versus control group). Next, we tested the effects of BA on AMPK phosphorylation in a well-established LKB1-deficient cell line, HeLa cells. HeLa cells show a lack of LKB1 expression. However, as shown in **Figure 6C**, BA still promoted remarkable AMPK activation in HeLa cells (all *p* < 0.01 versus control group), indicating that BA-activated AMPK was independent of LKB1. Next, we investigated whether CaMKKβ was required for BA activation of AMPK. We observed that BA-activated AMPK phosphorylation was significantly attenuated by the CaMKKβ inhibitor STO-609 in LPS-treated BV-2 microglial cells (**Figure 6D**). Collectively, by using both pharmacological and siRNA approaches, we showed that BA activated microglial AMPK in a CaMKKβ-dependent manner.

### BA Promoted Microglia M2 Polarization via CaMKKβ-Dependent AMPK Activation

**FIGURE 7:**
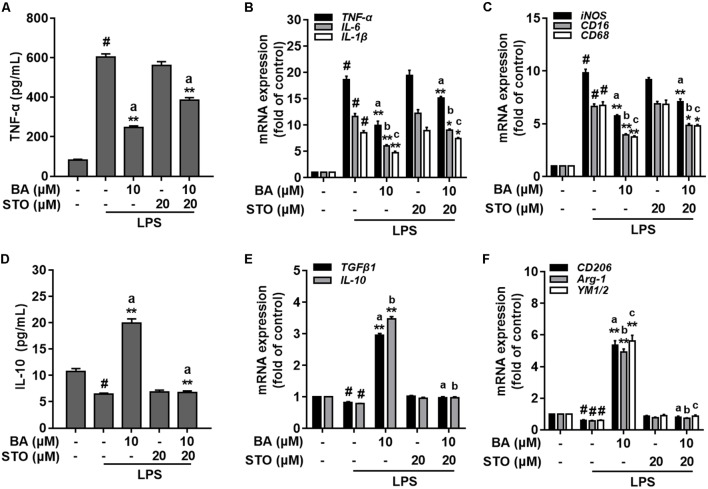
CaMKKβ-dependent AMPK activation is involved in BA-mediated microglia polarization in BV-2 microglial cells. BV-2 cells were pretreated with CaMKKβ inhibitor STO-609 (STO) for 0.5 h, and then with BA for another 1 h, followed by exposure to LPS stimulation for 6 or 24 h. **(A)** STO reversed BA inhibition of LPS-induced TNF-α production. **(B,C)** STO attenuated BA inhibition of LPS-induced mRNA expression of TNF-α, IL-1β, IL-6, iNOS, CD16, and CD68. **(D)** STO abolished BA-enhanced M2 marker protein IL-10 release into culture media. **(E,F)** STO abolished BA-enhanced mRNA expression of TGFβ1, IL-10, CD206, Arg-1, and YM1/2. Data are presented as means ± SEM of three independent experiments in triplicate. Control group was untreated cells. ^#^*P <* 0.01, versus control group; ^∗^*P <* 0.05 and ^∗∗^*P <* 0.01, versus LPS-treated group. Two columns sharing the same letter are significantly different (*P <* 0.05).

**FIGURE 8:**
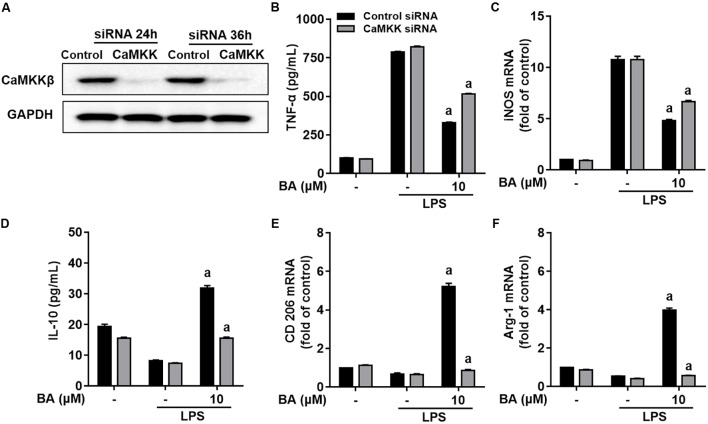
Knockdown of CaMKKβ by siRNA attenuated BA-promoting effects on M2 polarization of BV-2 microglial cells. Cells were first transfected with 45 nM CaMKKβ siRNA for 24 h, and then treated with BA for 1 h, followed by exposure to LPS stimulation for 6 or 24 h. **(A)** Transfection for 24 h was able to decrease the expression of CaMKKβ protein. **(B,C)** CaMKKβ knockdown decreased BA-mediated inhibition of TNF-α production, and mRNA expression of TNF-α and iNOS in LPS-stimulated BV-2 cells. **(D–F)** CaMKKβ knockdown abolished BA-enhanced IL-10 release and mRNA expression CD206 and Arg-1. Data are presented as means ± SEM of three independent experiments in triplicate. Control group was that treated with control siRNA but not BA or LPS. Two columns sharing the same letter are significantly different (*P <* 0.05).

We further investigated whether CaMKKβ was functionally required for BA’s promoting effects on M2 microglial polarization. First, BA-induced inhibition of TNF-α expression, and gene expression of TNF-α, IL-6, IL-1β, iNOS, CD16, and CD68 were in part attenuated by the CaMKKβ inhibitor, STO-609 in BV-2 cells (**Figures 7A–C**, all *p* < 0.01 versus the LPS-treated group). Moreover, STO-609 entirely abolished IL-10 protein expression, which was enhanced by BA treatment in BV-2 cells (**Figure 7D**, *p* < 0.01 versus BA and LPS-treated group). In addition, STO-609 entirely abolished BA-induced upregulation of IL-10, TGFβ1, CD206, Arg, and Ym1/2 in LPS-treated BV-2 (**Figures 7E,F**, all *p* < 0.01 versus BA and LPS-treated group). Consistently, CaMKKβ siRNA also displayed similar effects on the expression of IL-10, CD206, and Arg-1 in BV-2 cells (**Figures 8A,D–F**), indicating that CaMKKβ knockdown also abolished the effects of BA on promoting microglia toward M2 polarization. On the other hand, even though CaMKKβ knockdown decreased BA-mediated inhibition of LPS-induced microglia M1 inflammation (upregulation of TNF-α and iNOS, as shown in **Figures 8A–C**), CaMKKβ siRNA did not completely abolish BA’s inhibitory effects on M1 inflammation. In conclusion, M2 polarization by BA involves CaMKKβ-dependent AMPK activation.

### BA Induced CaMKKβ/AMPK Phosphorylation and Promoted Microglia M2 Polarization in the LPS-Induced Mouse Model

**FIGURE 9:**
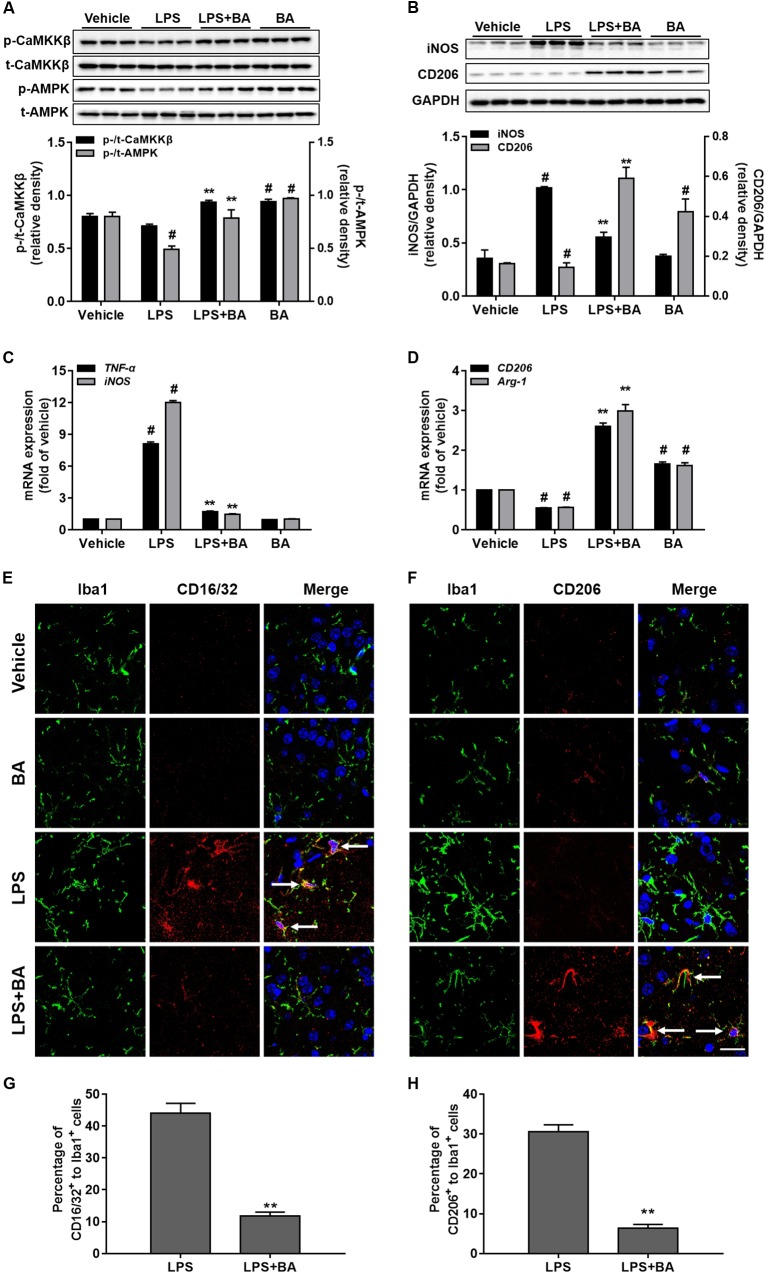
Effects of BA on CaMKKβ/AMPK activation and microglia M2 polarization in the LPS-induced mouse model. The animals were treated with or without BA (30 mg/kg, i.p., once per day) for 4 days, and then injected intraperitoneally with LPS (1 mg/kg) 1 h after the last BA treatment; 6 h after LPS stimulation, animals were sacrificed and brains were then obtained and further isolated for assay. **(A)** BA significantly increased the phosphorylation of CaMKKβ and AMPK in the cerebral cortex of mouse brain. **(B)** BA obviously inhibited iNOS expression, but enhanced M2 marker protein CD206 expression. **(C,D)** BA significantly inhibited the mRNA expression of TNF-α and iNOS, whereas it increased mRNA expression of CD206 and Arg-1. **(E,F)** Double staining of Iba1 (green) with CD16/32 (red) or CD206 (red) in slices section of cerebral cortex. Nuclei are counterstained with DAPI (blue). Scale bar, 20 μm. Arrows indicate obvious CD16/32 or CD206 positive microglia. Representative images were obtained from one set of experiments, and the three experiments were performed independently. **(G,H)** The quantity of the percentage of CD16/32^+^ to Iba1^+^ cells and CD206 to Iba1^+^ cells. Data are presented as means ± SEM. Control group was untreated cells. ^∗^*P <* 0.05 and ^∗∗^*P <* 0.01, versus LPS-treated group.

It is well known that LPS triggers robust immune responses and intraperitoneal injection of LPS is widely used to induce systemic inflammation and behavior responses in rodent models (Henry et al., 2008; Xu et al., 2015). As shown in **Figures 9A,B**, compared with the control and LPS group, treatment with BA (30 mg/kg) significantly increased phosphorylation of CaMKKβ and AMPK in the cerebral cortex. LPS injection significantly induced the expression of TNF-α and iNOS in mRNA and reduced CD206 and Arg-1 mRNA expression (**Figures 9C,D**, all *p* < 0.01 versus vehicle group). Conversely, BA (30 mg/kg) administration suppressed LPS-induced mRNA expression of TNF-α and iNOS, and promoted enhanced M2 gene CD206 and Arg-1 mRNA expression (all *p* < 0.01 versus BA and LPS-treated group).

The morphology of microglia cells in the resting condition most frequently showed a small and ovoid shape. Most of the LPS-stimulated microglia cells had morphology changes with large and flat shape, while BA can inhibit activation of the microglia by LPS, as evidenced by typically resembling morphology that was seen in the control group (**Figures 9E,F**). The expression of the M1 marker CD16/32 was lower in Iba1^+^ microglia in LPS + BA treated group than that in LPS group (**Figures 9E,G**). Moreover, the co-expression levels of the M2 marker CD206 and Iba1 were higher in the LPS + BA group compared with the LPS group (**Figures 9E,H**).

### BA Had No Effect on Phagocytic Capacity in LPS-Activated BV-2 Microglial Cells

**FIGURE 10:**
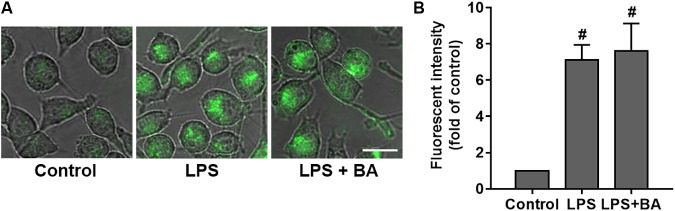
BA had no effect on phagocytic capacity in LPS-activated BV-2 microglial cells. BV-2 cells were treated with BA for 1 h and then incubated with LPS for another 24 h. **(A)** Effect of BA on phagocytic capacity in LPS-activated BV-2 cells. Scale bar, 20 μm. **(B)** The relative fluorescence intensity of the cells. Data are presented as means ± SEM of three independent experiments in triplicate. Control group was untreated cells. ^#^*P <* 0.01, versus control group.

The phagocytic ability of microglia following treatment with LPS with or without BA was comparatively examined through quantification of their capacity to phagocytose IgG FITC-conjugated latex beads. As shown in **Figure 10**, compared to control untreated cells, LPS stimulation of BV2 microglia to an M1 phenotype enhanced their ability to phagocytose fluorescent beads (*p* < 0.01). Interestingly, we observed that BA treatment did not impair LPS-enhanced phagocytic capacity in BV-2 cells (*p* < 0.01 versus LPS-treated group).

## Discussion

Inflammatory responses are inevitable and important pathological processes in several kinds of neurological diseases (Ransohoff and Perry, 2009). Microglia-mediated neuroinflammation and subsequent neurological damage contribute to several neurodegenerative diseases, like multiple sclerosis (MS), Alzheimer’s disease, and Parkinson’s disease (Ransohoff and Perry, 2009; Gemma, 2010; Dhama et al., 2015). Similar to macrophages, microglial cells show plasticity, and can polarize into different functional phenotypes in response to various microenvironmental disturbances. There are two two extremes: the pro-inflammatory M1 phenotype that induces pro-inflammatory cytokine release and exacerbates neuronal disorders, and the anti-inflammatory M2 phenotype that confers neuroprotective effects via enhancing the expression of genes involved in inflammation resolution, immunomodulation, homeostasis, scavenging, angiogenesis, and wound healing (David and Kroner, 2011; Saijo and Glass, 2011; Joseph and Venero, 2013; Mantovani et al., 2013). The pro- and anti-inflammatory responses need to be balanced to prevent the potential detrimental effects of a prolonged, unregulated inflammation. Thus far, the evidence presented for inflammation in neurodegenerative diseases points to an uncontrolled and prolonged M1-activated state that contributes to additional neuronal damage (David and Kroner, 2011; Saijo and Glass, 2011; Joseph and Venero, 2013; Mantovani et al., 2013). However, simply inhibiting inflammation by suppressing M1 activation would likely not confer overall benefits based on previous non-steroidal anti-inflammatory drug and AD anti-inflammatory prevention trial studies (Becker et al., 2011; Hu et al., 2015). Microglia activation states in neurodegeneration may need to be specifically treated by simultaneously attenuating M1 responses and promoting M2 responses. Inhibiting the M1 phenotype while stimulating the M2 phenotype has been suggested as a more viable potential strategy for the treatment of neuroinflammatory disorders (Hu et al., 2015). For instance, two agents approved for MS therapy, glatiramer acetate and β-interferon, have demonstrated not only M1-inhibiting actions, but also promote a balance between M1 and M2 cells (Burger et al., 2009; Kieseier, 2011). However, most of the compounds reducing neuroinflammation simply suppress M1 phenotype microglia, and few compounds have been demonstrated to promote the polarization of microglia to the M2 phenotype (Burger et al., 2009; Kieseier, 2011; Huang et al., 2017). BA is a widely used natural product and its anti-inflammatory properties are well known (Saaby and Nielsen, 2012; Wan et al., 2013; Ju et al., 2015; Lingaraju et al., 2015; Kim et al., 2016; Laavola et al., 2016). Moreover, BA was able to decrease pro-inflammatory cytokine release and matrix metalloproteinase expression in LPS-stimulated microglial cells, indicating a definite anti-neuroinflammatory role of BA in CNS (Lee et al., 2011; Wan et al., 2013). Therefore, it is of significance to test the role of BA in the regulation of microglia polarization, and to further explore the possible molecular mechanisms. Generally, in activated microglia, M1 or M2 polarization is characterized and distinguished by expression of pro- and anti-inflammatory genes and proteins, respectively (David and Kroner, 2011; Saijo and Glass, 2011; Joseph and Venero, 2013; Mantovani et al., 2013). LPS is a classical Toll-like-receptor-4 agonist that can not only polarize microglia into the M1 pro-inflammatory phenotype, thus inducing inflammatory responses, but also decrease the expression of M2 anti-inflammatory markers, thus attenuating inflammation (David and Kroner, 2011; Saijo and Glass, 2011; Joseph and Venero, 2013; Mantovani et al., 2013). Therefore, the LPS-stimulated microglial models were widely used to explore the regulation and mechanism of microglial polarization (Henry et al., 2008; Xu et al., 2015). First, we confirmed that BA treatment could inhibit LPS-induced morphological changes of activated microglia in BV-2 cells. The current data also indicated that BA could significantly promote microglial polarization toward the anti-inflammatory M2 phenotype in LPS-stimulated BV-2 microglial cells. Recent studies have indicated the importance of the phagocytic activity of microglia in the injured CNS, in which the phagocytosis of dead neurons is crucial for recovery and promotes axon regeneration and restoration of the microenvironment (Cheon et al., 2017; Paetau et al., 2017). Previous study reported a phenomenon that the important role of classically activated M1 microglia, phagocytosis and debris clearance, was retained in the converted M2 phenotype microglia (Cheon et al., 2017; Paetau et al., 2017). Interestingly, in the current study, we also found that BA did not impair the phagocytic activity of activated microglia. Furthermore, the cellular results were confirmed by the results of the systemic LPS injection mouse model. The *in vivo* data also indicated that BA remarkably suppressed M1 phenotype marker expression and promoted polarization of microglial cells into the M2 phenotype, which might contribute to BA-induced neuroprotective effects on neuroinflammation. Taken together, the current data suggested that BA could promote microglia polarization toward the M2 anti-inflammatory phenotype, which at least in part contributes to BA-mediated neuroprotection.

Accumulating evidence indicates that cellular energy metabolism not only participates in inflammatory responses but also regulates the conversion of functional phenotypes in immune cells (O’Neill and Hardie, 2013). The AMPK is an evolutionarily conserved intracellular energy metabolism sensor that has a central role in maintaining energy homoeostasis (Amato and Man, 2011; O’Neill and Hardie, 2013). AMPK is one of the most important endogenous neuroprotective molecules against inflammatory responses (Sag et al., 2008; Bai et al., 2010). AMPK not only plays a crucial role in suppressing inflammatory cytokines and mediators in activated microglia, but also promotes microglial polarization toward the M2 stage (Amato and Man, 2011; O’Neill and Hardie, 2013; Hussein et al., 2015; Liu et al., 2016; Velagapudi et al., 2017). Therefore, AMPK has been considered as an attractive target for the treatment of inflammatory diseases (Amato and Man, 2011; O’Neill and Hardie, 2013). Recently, several reports also demonstrated that BA could activate AMPK pathways in various *in vitro* and *in vivo* models (Jin et al., 2016; Silva et al., 2016; Xie et al., 2017). On the other hand, accumulating data indicated that the anti-inflammatory properties of some widely known anti-inflammatory agents, like salicylate, are also strongly associated with the activation of the AMPK signaling pathway (Hawley et al., 2012). Moreover, some synthetic and natural compounds that could induce AMPK also confer anti-inflammatory actions and promote M2 polarization of microglia (Lu et al., 2010; Lee and Kim, 2014; Mancuso et al., 2014; Zhou et al., 2014). Hence, it was of interest to investigate the relationship between anti-neuroinflammatory effects and AMPK activation by BA. In the current study, we first found that, in the absence of LPS, BA robustly increased AMPK phosphorylation in BV-2 microglial cells. LPS, usually served as a potent M1 inducer, was also observed to significantly decrease AMPK activity in microglia cells. However, BA not only attenuated LPS-induced AMPK activation reduction but also raised AMPK phosphorylation up to the level several-folds that of the baseline. These current data are consistent with previous reports showing that BA-induced AMPK activation conferred protection in endothelial cells and mouse models (Lingaraju et al., 2015; Jin et al., 2016). Next, we confirmed that BA’s effects on inhibiting M1 polarization and promoting M2 polarization were dependent on AMPK activation using the AMPK inhibitor, compound C, and AMPKα siRNA to block AMPK. Of note, pre-treatment with an AMPK inhibitor or AMPKα siRNA abolished BA-mediated regulation of M2 markers in LPS-stimulated BV-2 cells. The current data also coincided with a previous publication, indicating that increased number of M2 microglia subtype population may enhance AMPK activity as a feedback mechanism (Sag et al., 2008). In addition, BA-enhanced AMPK phosphorylation was also found in the cerebral cortex in an LPS-stimulated mouse model. Based on the above-mentioned results, we suggested that BA’s beneficial effects in the mouse model might be, at least in part, mediated by AMPK activation. Therefore, the current results suggested that BA-promoted microglia M2 polarization was mediated by the activation of AMPK signaling pathways.

It is well known that AMPK activation is dependent on the phosphorylation of Thr172 in the activated loop (Amato and Man, 2011; O’Neill and Hardie, 2013). AMPK can be activated by multiple mechanisms (Mounier et al., 2013; O’Neill and Hardie, 2013). For instance, AMPK activation could be regulated by its major upstream AMPK kinase, LKB1, in response to energy fluctuations (Amato and Man, 2011; O’Neill and Hardie, 2013). Correspondently, certain AMPK activators, like metformin, act through respiratory chain inhibition and indirectly activate AMPK; furthermore, LKB1 is indispensable for metformin-induced activation of AMPK (Owen et al., 2000). In addition to LKB1, CaMKKβ is another upstream kinase that can directly phosphorylate AMPK on Thr172 in response to changes in intracellular calcium (Amato and Man, 2011; O’Neill and Hardie, 2013). Therefore, further experiments were designed to investigate the role of BA in these two upstream kinases (Kieseier, 2011; Hawley et al., 2012). First, we found that BA induced CaMKKβ phosphorylation, but not of LKB1, in BV-2 microglial cells. Next, we found that AMPK was also phosphorylated in LKB1-deficient HeLa cells (Racioppi et al., 2012), which further suggested that BA-mediated AMPK activation was not dependent on LKB1 activity. On the other hand, the application of the pharmacological CaMKKβ inhibitor, STO-609, significantly abolished BA-induced AMPK phosphorylation, all of which indicated that CaMKKβ is indispensable for BA-mediated AMPK activation. The role of BA in regulating CaMKKβ-dependent activation of AMPK in microglial cells was consistent with previous reports showing that BA could induce CaMKKβ-dependent, but not LKB1-dependent AMPK activation in endothelial cells and in a mouse model of non-alcoholic fatty liver disease. Although the detailed mechanisms of CaMKKβ’s action in microglia-mediated inflammation still remain unclear, it appears that CaMKKβ confers anti-inflammatory effects in microglial cells in several models (Racioppi et al., 2012). There were several reports indicating that some agents, including berberine, telmisartan, and H_2_S donors, induce CaMKKβ-dependent AMPK activation and promote M2 polarization of microglia via the CaMKKβ/AMPK pathway (Lu et al., 2010; Zhou et al., 2014; Xu et al., 2015). Based on the current results, we suggest that BA promoted M2 polarization of microglia via CaMKKβ-dependent AMPK activation in microglia cells. However, further studies are warranted to determine whether BA directly activates CaMKKβ or indirectly regulates CaMKKβ via other targets.

In the current study, we also found BA to be a potent AMPK activator. Activation of the AMPK signaling pathway has considerable therapeutic potential in several neuroinflammation-associated diseases (Russo et al., 2013). Therefore, BA could be further exploited as a novel agent for treating these disorders. Moreover, most of the drugs targeting AMPK activation in current clinical use act mainly via mitochondrial inhibition, increasing AMP/ATP ratios during AMPK activation (Cunniff et al., 2016; Xu and Ash, 2016). In contrast, no agents have been shown to directly mediate AMPK activation or indirectly regulate upstream kinases, other than LKB1, in human studies (Cunniff et al., 2016; Xu and Ash, 2016). Our results interestingly showed that BA-mediated AMPK activation was dependent on CaMKKβ, but not LKB1, in microglial cells. Whether BA-induced activation of AMPK is also dependent on an increase in the AMP/ATP ratio, or whether CaMKKβ activation alone for the activation of AMPK by BA, will be investigated in our future studies. Furthermore, AMPK is an upstream kinase that regulates PPARγ activation, indicating that the relationship between these two systems is context-dependent (Yazawa et al., 2010; He et al., 2011). It is well-estbalished that AMPK and peroxisome PPARγ are closely integrated in microglia cells, showing similar effects such as inhibiting pro-inflammatory gene expression and regulating energy metabolism balance (Yazawa et al., 2010; He et al., 2011). Previous reports also demonstrated that BA inhibited IL-1β-induced inflammation via activating PPARγ in osteoarthritis chondrocytes. Therefore, it is also interesting to further explore the role of BA in regulating the PPARγ pathway in microglia cells.

## Conclusion

Our study demonstrated that BA promoted microglia polarization toward the M2 phenotype via CaMKKβ-dependent AMPK activation. Therefore, the current results supported the notion that BA treatment could confer anti-neuroinflammatory actions and might have considerable value as a therapeutic agent against neuroinflammatory diseases.

## Ethics Statement

Anesthesia will be done when there is an invasive operation or before sacrifice. The Animal sacrifice was conducted via the animal anesthesia system using carbon dioxide/oxygen gas. Enough food and clean water were ensured. Enough space was given to the animal to avoid over-crowding. During animal handling, the procedure should be mild and quiet to prevent over-reaction and harm to the animals.

## Author Contributions

MH, CH, and SL conceived and designed the study. CL, CZhang, HZ, YF, and FT performed the experiments. CL, DM, CZhao, and SL drafted the manuscript.

## Conflict of Interest Statement

The authors declare that the research was conducted in the absence of any commercial or financial relationships that could be construed as a potential conflict of interest.

## Abbreviations

ACC: acetyl-CoA carboxylase
AMPK: AMP-activated protein kinase
Arg-1: arginase-1
BA: betulinic acid
CaMKKβ: calmodulin-dependent protein kinase kinase β
CD16: cluster of differentiation 16
CD68: cluster of differentiation 68
CD206: mannose receptor
CNS: central nervous system
COX-2: cyclooxygenase-2
IL-1β: interleukin-1β
IL-6: interleukin-6
iNOS: inducible nitric oxide synthase
LKB1: liver kinase B1
LPS: lipopolysaccharide
MTT: 3-(4,5-dimethylthiazol-2-yl)-2,5-diphenyltetrazolium bromide
NO: nitric oxide
PPAR-γ: peroxisome proliferator-activated receptor-γ
ROS: reactive oxygen species
siRNA: small interfering RNA
TGFβ: transforming growth factor β
TNF-α: tumor necrosis factor-α
Ym-1/2: chitinase 3-like 3
