# Supplementary material for: Inhibitory Effects of Betulinic Acid on LPS-Induced Neuroinflammation Involve M2 Microglial Polarization via CaMKKβ-Dependent AMPK Activation
Source: Front Mol Neurosci. 2018 Apr 3;11:98. doi: 10.3389/fnmol.2018.00098 (PMC5891622; doi:10.3389/fnmol.2018.00098)
Supplement: Supplementary file 1 [file Table_1.DOCX]

**Supplementary Table 1**

**Supplementary Table 1. Primers for qPCR**

| **Gene** | **Forward primer (5’→3’)** | **Reverse primer (5’→3’)** |
| --- | --- | --- |
| TNF-α | CCTATGTCTCAGCCTCTTCT | CCTGGTATGAGATAGCAAAT |
| IL-1β | GGCAACTGTTCCTGAACTCAACTG | CCATTGAGGTGGAGAGCTTTCAGC |
| IL-6 | CCACTTCACAAGTCGGAGGCTT | CCAGCTTATCTGTTAGGAGA |
| iNOS | CAAGAGTTTGACCAGAGGACC | TGGAACCACTCGTACTTGGGA |
| IL-10 | ATAACTGCACCCACTTCCCA | GGGCATCACTTCTACCAGGT |
| TGF-β1 | CAACAATTCCTGGCGTTACCTTGG | GAAAGCCCTGTATTCCGTCTCCTT |
| CD206 | CTTCGGGCCTTTGGAATA AT | TAGAAGAGCCCTTGGGTTGA |
| Arg-1 | GTGAAGAACCCACGGTCTGT | GCCAGAGATGCTTCCAACTG |
| YM1/2 | CAGGGTAATGAGTGGGTTGG | CACGGCACCTCCTAAATTGT |
| β-actin | AGCCATGTACGTAGCCATCC | GCTGTGGTGGTGAAGCTGTA |
| GAPDH | ATGTACGTAGCCATCCAGGC | AGGAAGGAAGGCTGGAAGAG |

**Supplementary Table 2**

**Supplementary Table 2. Sequence of target gene siRNA**

| **Gene** | **Sense strand (5′–3′)** |
| --- | --- |
| AMPKα siRNA | GAGAAGCAGAAGCACGACGTT |
| CaMKKβ siRNA | CAGGAGAUUGCUAUCCUCAAATT |
| Control siRNA | UUCUCCGAACGUGUCACGUTT |
